# Supplementary material for: Centralized Intake Models and Recommendations for Their Use in Non-Acute Mental Health Services: A Scoping Review
Source: Int J Environ Res Public Health. 2023 May 8;20(9):5747. doi: 10.3390/ijerph20095747 (PMC10177908; doi:10.3390/ijerph20095747)
Supplement: Supplementary file 1 [file ijerph-20-05747-s001.zip › ijerph-2318498-supplementary.pdf]

International Journal of
Environmental Research and Public Health

MDPI

Supplementary Files

Table S1. System challenges and objectives for implementing CI (Centralized intake) models.

**Author (ref)**	**System challenge**	**Objectives/modification of the CI model**
Substance use programs		
Levie, Claxton and Barnes [4]	Frustration and misunderstanding among staff of agencies that worked under the umbrella of the Metro Interagency Drug Abuse Program [MIDAP] in Omaha	To improve working relationships between the different drug treatment programs<br>To make the most appropriate treatment options immediately available to clients
Scott, Sherman, Foss, Godley and Hristova [22]	Not specifically reported	To implement a standardized assessment process<br>To decrease waiting times to access assessment and treatment through maintenance of a centralized waiting list<br>To develop and implement a management information system (MIS) to support CI client tracking and reporting functions<br>To improve the extent to which co-occurring or ancillary problems are identified and addressed through referrals to other health and human service providers
Woods, Klingemann and Guydish [23]	Not specifically reported	To make better use of the existing capacity of services where clients could be quickly directed to the most appropriate service, thereby improving access and outcomes
Berends and Hunter [5]	Existing services did not comprise a system and clients' treatment needs were rarely matched to treatment content. Challenges included weak infrastructure, inconsistent service quality, limited access to treatment, and inadequate service coordination and continuity.	Not specifically reported
Mental health services		
Sawyer and Moreines [20]	Young people with mental illness were found to have multiple problems and needs that placed a significant burden on their families and services	To concurrently identify children's specific needs through synthesis of information from different agencies.
Melathopolous and Cawthorpe [12]	After several Canadian public hospital systems merged, a CI system and associated regional access<br>and intake system were developed and implemented for child and adolescent mental health services.	The model of care was made episodic. Once registered and assessed and/or treated, patients could be transferred within regional services, or be discharged after treatment.<br>Individuals could re-enter scheduled or urgent community or hospital-based emergency and inpatient services under a new registration.
Isaacs, Sutton, Dalziel and Maybery [6], Isaacs, Beauchamp, Sutton and Kocaali [36]	Difficulties faced by individuals with severe mental illness in accessing multiple services resulting in their falling through the cracks in the system	To facilitate a better integrated mental health care system by improving referral pathways, strengthening partnerships between the different services that would work together and promoting a community-based recovery model
Northwestern Melbourne PHN [40]	The rapid increase in mental health problems in the population and the resultant increase in demand for services	To support persons of all ages with their mental health problems
Schuble, Graham and Covington [28]	Americans living in Georgia who were in crisis or in need of behavioural health services had only two options: 1. To find a provider on their own or 2. To call one of 25 "single-point-	To establish a state-wide toll-free crisis and access line, called the Georgia Crisis and Access Line (GCAL) that provides a "no wrong door" approach to access, offering telephonic crisis

	of-entry" phone lines serving portions of Georgia to get provider information. Both options required individuals to make their own appointments, which often took time. Many ended up in hospital emergency rooms, state hospitals or local jails. This significantly increased the cost of care, or no care at all.	intervention, 24/7 service scheduling with consumer choice, real-time data and reporting for strategic planning, and a new degree of consistency and transparency.
Hutt-MacLeod, Rudderham, Sylliboy, Sylliboy-Denny, Liebenberg, Denny, Gould, Gould, Nossal, Iyer, Malla and Boksa [37]	The community of Eskasoni First Nation suffered the loss of a number of youth by suicide and accidental drug overdose. The resulting negative news headlines caused emotional, mental and economic suffering to the population. Almost every young person in the [Eskasoni] community was negatively impacted with several having contemplated suicide.	To improve ease of contact through social media and other modalities<br>Formerly siloed community mental health services, crisis services and case management services were amalgamated and co-located into a united and integrated team under one director
Duncombe [26]	No records describing CI processes for rural community health services for counselling.	To review CI processes for rural community health services for counselling
Residential care services		
Hamm and Callahan [8]	The home health care provider community was facing several challenges such as the increased demand for services, increased pressure owing to decreased utilisation of services per case, decreased reimbursement and increased financial and clinical accountability.	To reduce costs of providing services and increase satisfaction among the users of the system (hospitals, physicians, patients, and insurance companies)
Mohr and Bourne [24]	An increasing demand for services, pressures from acute care, the need to reconfigure residential care facilities and increasingly complex client needs within the community care programs.	To facilitate more effective screening in the intake process which warranted a clinical role in the intake process.
City of Toronto [42]	The lack of adequate emergency and other Homelessness services.	To refer callers experiencing homelessness to available emergency accommodation, including referrals to City-operated or partner-operated shelters and 24-hour respite sites
Cardiac services		
Bungard, Smigorowsky, Lalonde, Hogan, Maier and Archer [25]	General Practitioners had difficulty accessing cardiology consultation. Booking a single consultation required multiple telephone calls owing to the variable availability of cardiologists and the uncertainty as to which cardiologist had the expertise for a particular patient. Many cardiologists at the University of Alberta Hospital were new faculty members, and hence were relatively unknown to general practitioners.<br>Triage for elective consults was often delegated to secretaries who could not triage patients without consultants. Consequently, patients often lacked appropriate testing when seen in consultation.<br>These problems contributed to unnecessary and costly repeat visits and dissatisfaction by patients and physicians alike. There was no system to inform the referring physician of	To establish a programmatic approach that systematically triaged patients, coordinated pre-consultation testing and implemented strategies for efficient information transfer.

	the test results or advice of the consultant in a uniform and timely manner.	
Family support services		
Early Childhood Iowa Quality Services and Programs Component group [30]	Professionals found it challenging to be up to date with what family support services were available in their community and which service best met the needs of the family.	To assist families determine services and supports that were best suited for their particular needs based on the referral
Arthritis services		
Barber, Patel, Woodhouse, Smith, Weiss, Homik, LeClercq, Mosher, Christiansen, Howden, Wasylak, Greenwood-Lee, Emrick, Suter, Kathol, Khodyakov, Grant, Campbell-Scherer, Phillips, Hendricks and Marshall [13]	Several patients with osteoarthritis and rheumatoid arthritis in Canada experience delays in access to care and treatment with many not receiving care within recommended benchmarks. The reasons for delays in care are complex and include a mismatch between supply and demand for specialist care in many regions. Inefficiencies at the level of referral and triage also lead to delays in care.	Not specifically reported
Marshall, Bischak, Zaerpour, Sharif, Smith, Reczek, Robert, Werle and Dick [44]	Delays in access to specialty care and elective total joint replacement surgery were impacting on quality of life of patients and resulting in increased costs for the health care system. With the ageing population and increasing rates of obesity, wait times were expected to rise without appropriate strategies to manage the waitlist.	Different wait time management strategies were utilized in a CI system for hip and knee replacement surgery
Damani, MacKean, Bohm, Noseworthy, Wang, DeMone, Wright and Marshall [7]	Long waiting times for care resulting in decline in health status and loss of confidence in the system.	To balance waiting time for hip or knee surgery across orthopaedic surgeons<br>To improve patient access by referring to the next available surgeon<br>To optimize the referral process by streamlining utilisation of resources and eliminating non-value added activities throughout the referral process.
Paediatric rehabilitation services		
Wittmeier, Restall, Mulder, Dufault, Paterson, Thiessen and Lix [9]	Paediatric rehabilitation services were provided to preschool children by six different service providers. Each provider had their own mandate and wait list. Children in need of rehabilitation services were often referred to multiple providers based on diagnosis rather than need for therapy, and there was little communication among and between service providers. Consequently, children could be waiting for therapy at multiple sites and in some cases, be receiving treatment from two or more therapists from the same profession. Physiotherapy was one of the first disciplines to start using the central intake system.	Not specifically reported.
Multiple community services		
Grampians Health Ballarat [43]	Not specifically reported	An access and referral management service for all referrals received for community programs such as post-acute care, the hospital admission risk program, regional continence Service, allied health services, aged care assessment service, palliative care, carer respite and support services

New South Wales Health. Southern NSW Local Health District [41]	Not specifically reported	A telephone and email based central intake service which receives and processes referrals for many different community health services across the Southern NSW Local Health District

Table S2. Characteristics of CI models.

**Author, (ref)**	**Initial engagement (Intake)**
Levie, Claxton and Barnes [4]	All new clients, whether they were self-referred or referred from the court or another agency, were sent to the central intake office. The Central intake office was staffed by two Intake workers who gathered preliminary social history as well as obtained data from previous treatment and scheduled a physical exam. The functions of the office later evolved to include tracking of every client from entry to departure with a 24-hour answering system
Hamm and Callahan [8]	Referral to the CI in the home health integrated delivery system was in the form of a phone call or fax and could be from any stakeholder. Demographics, insurance carrier and number as well as specific referral information were used to register the patient into the system by Intake coordinators (customer service representatives). Once all documentation was verified with the insurance provider, the referral was forwarded to the Central Intake Department where a Nurse case manager (NCM) was assigned to the case
Mohr and Bourne [24]	Initial engagement was via a single access point to Community and Residential Care for information, referral and services. Clerical staff conducted the intake.
Bungard, Smigorowsky, Lalonde, Hogan, Maier and Archer [25]	Referrals were received by the CI office from General Practitioners, emergency departments, or specialty clinics. The secretary reviewed the referral, requested additional information if required, contacted the patient for additional information and provided clinic information.
	Initial engagement (Intake) and referral
Levie, Claxton and Barnes [4]	In certain situations, clients were referred immediately after intake. For instance, juvenile marijuana offenders were referred for drug education courses without further screening or assessment.
City of Toronto [42]	The 24/7 central intake line was manned by central intake case workers who referred callers experiencing homelessness to available emergency accommodation, including referrals to City-operated or partner-operated shelters and 24-hour respite sites. They also supported callers through warm transfers to specialized services and respond to requests for information about other services and programs that may be seen as connected to homelessness response, such as detox programs, primary and mental health care supports, refugee services and legal services.
New South Wales Health, [41]	Community Health Central Intake Service is a telephone and email-based service which receives and processes referrals for many different community health services across the Southern NSW Local Health District.
Grampians Health, Ballarat, [43]	Central Intake is the access and referral management service for all referrals received for community programs. Intake is by e-referral or faxing the referral form.
	Initial engagement (Intake) combined with screening or assessment and referral
Schuble, Graham and Covington [28]	Georgians experiencing crisis or in need of behavioural health services called a 24/7 toll-free number. Licensed clinical staff undertake first-line triage and emergency/crisis support. Other support staff manage caller access and referrals to routine or non-emergency services.
Isaacs, Sutton, Dalziel and Maybery [6]<br>Isaacs, Beauchamp, Sutton and Kocaali [36]	Clients with severe mental illness either self-referred or were referred by family members or General Practitioners GPs or community organizations to a designated intake service. Clients were accepted into the initiative if they satisfied the specific criteria. Accepted clients were then referred to appropriate agencies called support facilitator organisations [SFO].
Sawyer and Moreines [20]	In the rural partnership model for children's mental health services, young people were referred to the centralized mobile intake team which was staffed by mental health, social service, probation, and school personnel who completed a comprehensive assessment. The intake team established a service package that comprehensively addressed the child's needs. When this involved multiple agencies, a lead agency was identified which assumed primary case management responsibilities for the child. All agencies involved in the care of the child agreed to work with that lead agency to meet the needs of the child and family. The Rural Partnership Model established a multi-agency case management committee which met monthly to discuss the child's ongoing progress and prevailing needs.
Woods, Klingemann and Guydish [23]	Data collected on intake included demographic characteristics, legal status, homelessness, and history of drug use and treatment. A CI episode generally consisted of an assessment and referral(s) to appropriate treatment and other services

Melathopolous and Cawthorpe [12]	Referral to the Central Intake Service is either by telephone or fax. When a call comes in from a professional (e.g., referring physician, therapist, school professional) or a parent, or occasionally the youth himself/herself, a telephone intake screening is completed at that time by the mental health clinician and reviewed for urgency. If the referral is deemed urgent, a call is placed to the client, or if deemed routine, a letter is sent to the parent or the guardian (including a community education schedule) and the referral source requesting a call back to complete a telephone screening when available. If a client or his/her family members are referred to a community organization, they are provided with the organization's contact information and direction on how to access this resource. If a client is referred to a Child and Adolescent Addiction, Mental Health and Psychiatry Program service, the client is placed on the wait list for that service in the regional access and intake system (RAIS) which is a centralised electronic record and registry available to all Child & Adolescent Addiction, Mental Health & Psychiatry Program CAAMHPP clinics in the region.
Marshall, Bischak, Zaerpour, Sharif, Smith, Reczek, Robert, Werle and Dick [44]	Triaging and assessment are combined by implementing three wait time strategies. 1. Prioritization based on urgency, 2. Sorting patients based on assessment for surgery and 3. Management of patients based on the assessed need for surgery.
Suter, Birney, Charland, Misfeldt, Weiss, Howden, Hendricks, Lupton and Marshall [31]	Referrals are received via fax or electronically. Referral is reviewed for completeness by the unit clerk and any missing information is requested<br>Data is entered into a Triage data base and referrals information attached. A registered nurse assesses urgency of referral and triaged. Accordingly; urgent patients are scheduled for immediate care, non-urgent patients are scheduled to see a musculoskeletal (MSK) screener or assigned to an appropriate wait list
Northwestern Melbourne Primary Health Network [40]	Intake is via a toll-free number that operates during working hours (8.30 to 5 pm). Experienced mental health professionals screen callers using select criteria and depending on their needs, connect them with suitable existing services, or to a Head to Health hub, either onsite or through telehealth or to specialist or acute mental health services, including emergency care.
Damani, MacKean, Bohm, Noseworthy, Wang, DeMone, Wright and Marshall [7]	Waitlist coordinators are responsible for intake, screening, allocation to surgeon and regular communication with surgical offices. All documentation used standardized criteria and questionnaires. Patients were given education materials to enable them to be well informed at the time of their consultation visit.
	Screening/ assessment in isolation
Levie, Claxton and Barnes [4]	Intake information was presented weekly to the Central Intake Committee [CIC] who recommended treatment. The CIC was made up of the two Intake workers (one of whom was the chairman of the committee), a consultant psychiatrist and representatives of agencies to which referrals would go for assessment.
	Screening/ assessment combined with referral
Scott, Sherman, Foss, Godley and Hristova [22]	The central intake unit (CIU) was responsible for conducting all intake assessments, managing a centralised waiting list, and referring clients to levels of care assessed to be most appropriate.
Mohr and Bourne [24]	Screening and referral to the most appropriate resource was conducted by a Central Intake Nurse.
Bungard, Smigorowsky, Lalonde, Hogan, Maier and Archer [25]	The nurse practitioner performed assessment, and collected additional information if required to make a provisional diagnosis. If the referral was accepted, it was triaged, pre-visit non-invasive testing arranged and clinic date arranged. Clinic appointment booked with most appropriate cardiologist, first available appointment, as applicable.
Isaacs, Beauchamp, Sutton and Kocaali [36]	At the SFOs, a care coordinator was assigned to a client who worked with them to develop a care plan based on their needs. Once a care plan was developed, the care coordinator (referred to as a support facilitator in the Partners in Recovery PIR program) brokered services from relevant agencies in accordance with the plan.
Hamm and Callahan [8]	In the home health integrated delivery system, the nurse case manager obtained approval from the patient and carers for treatment, confirmed financial responsibilities with them, assigned the patient to the appropriate treating team, or clinic and updated the referrer on the treatment plan. The NCM also tracks the patient progress and coordinates discharge from the system
Levie, Claxton and Barnes [4]	Almost all clients are referred to one of the agencies in the network and when appropriate, to other drug and alcohol or social agencies as well. Some clients, particularly younger offenders were referred directly to specialized agencies without taking a preliminary history

Table S3. Benefits and challenges of utilisation of a CI service model for clients, service providers and the health system.

**Author, (ref)**<br>**CI model**	**Clients**	**Service providers**	**Health system**
Substance abuse programs			
Levie, Claxton and Barnes [4]<br>1. Initial engagement with 24-hour answering system<br>2. Assessment by Central Intake Committee<br>3. Referral to service providers<br>4. Tracking of clients from entry to departure	Benefits<br>Clients and the community knew where to go for help when there was a drug abuse problem<br><br>Challenges<br>Not specifically reported	Benefits<br>Service provider agencies came to have realistic expectations from each other and the referring consultant.<br><br>Challenges<br>High turnover of members of the Central Intake Committee which meant that staff had to learn their roles and get acquainted with that of each other.<br>Medical decisions such as the choice and dosage of methadone were usually made prior to the CIC meeting. In such cases, the psychiatrist would recommend changes when necessary	Benefits<br>Consistency of approach resulted in a better understanding among the Central Intake Committee [CIC] of which programs worked best for which clients. Since the CIC had flexibility in making plans for clients such as assigning clients to more than one agency, the committee members came to have realistic expectations from each other and the consultant. The psychiatrist only served as a consultant and not the leader of the group and offered psychiatric and medication advice in a 'non-threatening manner' to the group. Having the same psychiatrist for a prolonged time gave the group more confidence and understanding.<br><br>Challenges<br>Not specifically reported
Woods, Klingemann and Guydish [23]<br>1. Multiple mode engagement alongside the existing system therefore serving as an alternative entry to the system<br>2. Assessment<br>3. Referral to service providers	Benefits<br>Several clients particularly those with a disability, and those more likely to be on probation or incarcerated at the start of the CI episode entered the system for the first time.<br><br>Challenges<br>Not specifically reported	Benefits<br>Health providers sometimes referred their own clients to the CIU for assessment and referral. This was reported to occur when providers had waiting lists or conducted an intake on a client who proved to be inappropriate for the services provided by that agency, or when clients completed a treatment program and needed referral for additional treatment.	Benefits<br>None reported<br><br>Challenges<br>Methadone treatment programs had consistently high demand, insufficient supply of treatment slots, and intractable waiting lists, The CIU was understood, by clients and by the community, to be ineffective in bridging barriers to treatment access. As a consequence, persons seeking methadone treatment became less likely to do so through the CIU. While a CIU can improve access to a network of services, the absence of treatment slots and funding could hamper the usefulness of CI.
Rohrer, Vaughan, Cadoret, Carswell, Patterson and Zwick [21]<br>1. Intake and assessment done by full-time counsellors followed by referral to the appropriate facility	Benefits<br>Not specifically reported<br><br>Challenges<br>Clients considered CI as 'two-stop shopping'; Clients lacked motivation to travel to another site for treatment; Clients may be unwilling to face the psychological barriers of waiting time, denial,	Benefits<br>Not specifically reported<br><br>Challenges<br>Not specifically reported	Benefits<br>Not specifically reported<br><br>Challenges<br>27 % of clients who used the CI attended treatment when compared to 48% of those who used regular intake; Clients who used CI were also less likely to complete treatment.

	embarrassment and inconvenience a second time.		
Schuble, Graham and Covington [28]<br>1. Intake is via a 24/7 toll-free number.<br>2. Licensed clinical staff undertake first-line triage and emergency/crisis support.<br>3. Other support staff manage caller access and referrals to routine or non-emergency services.	Benefits<br>Cut average patient time for intake to services by up to 60 percent<br>Removed barriers to service access, simplified the appointment process, and reduced patient wait times for service.<br><br>Challenges<br>None reported	Benefits<br>Not specifically reported<br><br>Challenges<br>Not specifically reported	Benefits<br>Saved over $70 million by diverting callers to community-based services, preventing the inappropriate use of emergency rooms and state hospitals.<br>Saved $1.2 million/year in operating costs compared to the previous 25-line system.<br>Cut the cost of handling a call while providing direct scheduling, linkage, and patient follow-up capabilities not available with other call-system alternatives.<br>Enforced state-wide consistency in the identification and use of appropriate and least-invasive treatment interventions.<br>Provided a continuing source of performance data that supports continuous system improvement.<br><br>Challenges<br>None reported
Home health integrated delivery system			
Hamm and Callahan [8]<br>1. Multiple mode initial engagement<br>2. Assessment by Nurse Case Manager<br>3. Referral to service providers	Benefits<br>Increase in customer satisfaction.<br><br>Challenges<br>The centralised telephone system was also overwhelmed during peak call times resulting in long wait times and frequent busy signals for customers.	Benefits<br>Not specifically reported<br><br>Challenges<br>Not specifically reported	Benefits<br>Cost effectiveness and better clinical decision-making processes and quality outcomes through coordination of services, and consistency of practice through the implementation of diagnosis-specific plans and pathways.<br>Centralization of intake and case management was found to reduce costs. It has also been found to be effective in providing more appropriate referrals, within and beyond the community programs.<br><br>Challenges<br>During the course of the first year of the home health integrated delivery system model, the original mission of the management service organization [MSO] changed from being a home health integrated delivery system with a broad range of services to the narrow focus of management of home health needs of patients. As a result of this shift, the very purpose of having CI no longer existed and so intake was subsequently returned to hospital departments.<br>Despite several efforts to correct the system, the situation did not improve with new challenges emerging.
Residential care service			
Mohr and Bourne [24]	Benefits	Benefits	Benefits

1. Initial engagement via a single access point to Community and Residential Care<br>2. Screening and referral by a Central Intake Nurse	Well received by all stakeholder groups<br><br>Challenges<br>Not specifically reported	Well received by all stakeholder groups<br><br>Challenges<br>Many conflicting priorities and opinions on staffing and posting.	Consistent, standardized approach to services, targeting client needs with the determination and prioritization of urgent need. One contact site for out of health service area referrals. New clinically led system is demonstrated to be more effective, providing more appropriate referrals within and beyond the community programs.<br><br>Challenges<br>The skills and experience required for the Intake Nurse were not adequately reflected in the classification. Mental Health Elder Services (Indigenous) withdrew due to competing pressures and increased funding was required for longer hours.
Cardiac care			
Bungard, Smigorowsky, Lalonde, Hogan, Maier and Archer [25]<br>1. Initial engagement by the secretary<br>2. Reviewed the referral, requested additional information if required<br>3. Provided clinic information.	Not specifically reported	Not specifically reported	Benefits<br>The first three years of the Cardiac EASE program was investigator-initiated. After this time, Cardiac EASE became an operational program funded by Capital Health, indicating recognition of the program's success by the health authority.
Homeless Help			
City of Toronto [42]<br>1. Initial engagement with 24-hour answering system<br>2. Referral to service providers	Lower call wait times, improved call answer rates.	Not specifically reported	Ability to respond to higher call volumes. Steps are underway to improve availability and better use of Central Intake data to improve quality of care.
Mental health services			
Sawyer and Moreines [20]<br>1. Referral to multi-professional centralised mobile intake team who complete a comprehensive assessment and established a service package involving multiple agencies that comprehensively addressed the child's needs. A lead agency is established.<br>2. A multi-agency case management committee meets monthly to discuss the child's ongoing progress and prevailing needs.	Benefits<br>Significantly improved access by simplifying the process of identifying needs and providing necessary services. It provided time savings for the child, and parents. It offered a service package that was more likely to meet most of the child's needs.<br><br>Challenges<br>Not specifically reported	Benefits<br>It could complete a more comprehensive and integrated assessment than individual services, with time savings for agencies involved.<br><br>Challenges<br>Not specifically reported	Benefits<br>The system allowed for modifications and improvement in the way it functioned.<br>The multi-agency case management committee provided the ongoing continuity of care and therefore avoided the disconnected, and ineffective determinations of a child's prevailing needs. The committee could also change service packages as needs changed or treatment plans proved unsuccessful.<br>Still in operation after 10 years of starting it<br><br>Challenges<br>There were initial challenges in establishing networks and getting the various agencies to agree to work together. Additionally, there were continuing challenges with a lack of child mental health clinicians in the regions

Melathopolous and Cawthorpe [12]<br>1. Multiple mode initial engagement and screening and triage by mental health clinicians<br>2. Referral to community organisation or Child and Adolescent Addiction, Mental Health and Psychiatry Program service.	Benefits<br>There were reductions in wait times and length of stay.<br><br>Challenges<br>Not specifically reported	Benefits<br>Not specifically reported<br><br>Challenges<br>Not specifically reported	Benefits<br>Successfully implemented an episodic care model that contributed to increased service capacity, albeit within limits.<br><br>Challenges<br>Per capita rate of utilisation increased albeit marginally. However, the model did not alter the unmet need of children and youth with a physician-diagnosed mental disorder.
Isaacs, Sutton, Dalziel and Maybery [6]<br>Isaacs and Firdous [38]<br>Isaacs, Beauchamp, Sutton and Kocaali [36]<br>Waks, Scanlan, Berry, Schweizer, Hancock and Honey [34]<br>1. Initial engagement through toll-free telephone number followed by assessment based on fixed criteria<br>2. Referral to care coordinating agencies<br>3. Care coordinators broker services from multiple agencies according to a care plan developed with the client.<br>4. Clients leave the program when their needs are met or when they choose to.	Benefits<br>Care was client driven; Clients felt valued and more confident in managing their illness; Clients moved from despair to hope and reported better quality of life;<br>Clients felt improved mental clarity, focus and order in life; They were able to leave the house and engage in positive activity; They reported having a better social life; and improved physical health.<br><br>Challenges<br>Poor outcomes were reported when engagement with clients was challenging and when clients had multiple and complex problems particularly when those involved child protection.	Benefits<br>There was a team approach to patient care;<br>No duplication of services;<br>A better understanding of clients' history and needs<br><br>Challenges<br>Presentations and repeated discussions at team meetings of local area mental health services were necessary to allay the fears and suspicions of mental health clinicians who initially believed that their roles were being usurped;<br>Care coordinators who joined the program late did not receive adequate training; There was an initial lack of acceptance of care coordinators who were from culturally and linguistically diverse backgrounds; Care coordinators who were not from the local area lacked knowledge of local services and work culture	Benefits<br>Not specifically reported<br><br>Challenges<br>Not specifically reported
Surgery for arthritis			
Marshall, Bischak, Zaerpour, Sharif, Smith, Reczek, Robert, Werle and Dick [44]	Benefits<br>Improving triage for urgent patients and enhancing the precision of screening by deferring more patients to medical management programs can reduce the demand on the surgical queue and consequently reduce wait time for these patients. Urgent patients	Benefits<br>Not specifically reported<br><br>Challenges<br>Not specifically reported	Benefits<br>Combining a prioritization strategy with a sorting policy allows the provision of beneficial care for less urgent patients while ensuring the urgent patients do not unnecessarily wait longer to receive theirs.<br><br>Challenges<br>The long-term solution to reduce wait times needs to consider a blend of

	were expected to have lower wait times in all stages of care in the alternative scenario than non-urgent patients.<br>Improved sorting of patients according to their assessed needs into different streams further improves the patient flow by reducing the wait time for surgical patients while providing beneficial non-surgical care for the rest.<br><br>Challenges<br>Not specifically reported		solutions addressing the patient journey across all stages of the disease and the care continuum. On its own, prioritization strategies improve the wait times of urgent patients but increase that of less urgent patients
Suter, Birney, Charland, Misfeldt, Weiss, Howden, Hendricks, Lupton and Marshall [31]<br>1. Initial referral received electronically or via fax by the unit clerk.<br>2. Data is entered into a triage data base<br>3. Registered nurse assesses urgency of referral and triaged for further appointments	Benefits<br>Not specifically reported<br><br>Challenges<br>Not specifically reported	Benefits<br>Not specifically reported<br><br>Challenges<br>Not specifically reported	Benefits<br>Not specifically reported<br><br>Challenges<br>Bottleneck developed due to lack of available rheumatologists; Lack of musculoskeletal case-management team for stable and non-urgent patients<br>Inadequate support for musculoskeletal screener
Damani, MacKean, Bohm, Noseworthy, Wang, DeMone, Wright and Marshall [7]	Benefits<br>Overall waiting time was reduced.<br>Patients retained their availability to choose their surgeon<br><br>Challenges<br>Not specifically reported	Benefits<br>Increased referral volumes to the next available surgeon<br><br>Challenges<br>Slow uptake due to low initial awareness<br>Slow uptake of standards due to slow dissemination	Benefits<br>Optimised referral process<br><br>Improved quality of data<br>More complete referrals<br><br>Challenges<br>Top-down approach resulted in initial resistance from surgical offices<br>Initial lack of clarity and understanding among stakeholders about goals, processes and expectations Increased work burden for surgical offices<br>New offices were overwhelmed<br>Lack of familiarity with new processes leading to confusion among surgery office staff.
Paediatric physiotherapy			
Wittmeier, Restall, Mulder, Dufault, Paterson, Thiessen and Lix [9]	Benefits<br>More equitable wait times based on priority of need<br>Clear, simpler processes for accessing services in the right location<br><br>Challenges<br>Not specifically reported	Benefits<br>Improved communication between therapists<br><br>Challenges<br>Not specifically reported	Benefits<br>Reduced service duplication of services<br>More accurate wait time data<br><br>Challenges<br>Not specifically reported
